# Accelerated Aging in Cyclophilin B–Deficient Mice Downstream of p21‐Cip1/Waf1

**DOI:** 10.1002/jbm4.10674

**Published:** 2022-09-09

**Authors:** Ying Zhang, Robert J. Pignolo, Richard J. Bram

**Affiliations:** ^1^ Department of Pediatric and Adolescent Medicine Mayo Clinic College of Medicine Rochester MN USA; ^2^ Department of Medicine, Division of Geriatric Medicine and Gerontology Mayo Clinic College of Medicine Rochester MN USA; ^3^ Robert and Arlene Kogod Center on Aging Mayo Clinic College of Medicine Rochester MN USA; ^4^ Department of Immunology Mayo Clinic College of Medicine Rochester MN USA

**Keywords:** AGING, CYCLOPHILIN B, P21

## Abstract

Loss of bone mass and strength is a common problem of advanced age in humans. Defective bone is also a primary finding in osteogenesis imperfecta (OI), a genetic condition most commonly caused by autosomal dominant mutations in the type I collagen genes. Although altered collagen has been proposed to correlate with cellular processes that underlie aging, the causal relationships between them in vivo have not yet been completely explored. Whether aging plays a promoting role in OI development or whether OI contributes to aging, also remains unknown. The *PpiB* gene encodes cyclophilin B (CypB), a prolyl isomerase residing in the endoplasmic reticulum required for normal assembly of collagen. Germline deletion or mutations of CypB in mice or humans cause autosomal recessive OI (type IX). Here, we show that mice lacking CypB develop early onset of aging‐associated phenotypes, including kyphosis, fat reduction and weight loss, as well as abnormal teeth, skin, and muscle. Elevated senescence‐associated beta‐galactosidase (SA‐β‐Gal) activity was observed in fat tissues and in bone marrow–derived multipotent stromal cells. Protein levels of the cyclin‐dependent kinase (cdk)‐inhibitor p21‐Cip1/Waf1, a well known senescence marker, were significantly elevated in CypB‐deficient primary cells and mouse tissues. Importantly, loss of p21 in CypB knockout mice attenuated SA‐β‐Gal activity and delayed the development of kyphosis. In addition, less adipose tissue depot and higher SA‐β‐Gal activity were observed in a second OI model, *Cola2* ^*oim*^ mutant mice. A potential upregulation of p21 was also revealed in a limited number of these mice. These findings suggest that some of the features in OI patients may be mediated in part through activation of the p21‐dependent pathway, one of which is closely associated with senescence and aging. This study provides new mechanistic insight into relationships between OI and aging and raises the possibility of using senolytics drugs to treat OI in the future. © 2022 The Authors. *JBMR Plus* published by Wiley Periodicals LLC on behalf of American Society for Bone and Mineral Research.

## Graphical abstract

Insufficiency of CypB induces abnormal collagen. It also selectively promotes senescence of multipotent precursor cells. Both factors trigger the upregulation of the senescence marker, p21, which is considered to be a probable driver in the cascade whereby CypB deficiency may accelerate organismal aging. Whether abnormal collagen can directly accelerate senescence of multipotent precursor cells remains to be further investigated.

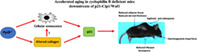

## Introduction

Osteogenesis imperfecta (OI) is a genetic bone disorder, typically presenting with symptoms of broken and deformed bones. The incidence of this disease is 1 in 10,000 to 20,000 people. The majority of cases are due to abnormalities of collagen, the most abundant protein in connective tissues that connect and support the whole body.^(^ ^1^ ^)^ Abnormal collagen can result from varied mutations in the type I collagen genes *COL1A1* or *COL1A2* or in genes that facilitate the stability, folding, secretion, or assembly of collagen trimers. Nineteen different genes have been reported to cause various types of OI, including *PpiB* (Cyclophilin B [CypB]) and its binding partners: prolyl‐3‐hydroxylase‐1 (*P3H1*) and cartilage‐associated protein (*CRTAP*).^(^ ^2^, ^3^, ^4^ ^)^

Cyclophilins are a highly conserved group of proteins that have specific affinity for the immunosuppressant drug cyclosporine.^(^ ^5^ ^)^ They have peptidyl‐prolyl isomerase activity that catalyzes the isomerization of peptidyl‐proline bonds. They can be found in most cellular compartments of most tissues.^(^ ^6^ ^)^ As an endoplasmic reticulum (ER) resident protein, CypB acts as a molecular chaperone and exerts various functions through its peptidyl‐prolyl cis‐trans isomerase activity in protein folding, secretion, and posttranslational modification.^(^ ^7^ ^)^ Multiple cellular functions of CypB have been identified, including immunosuppression,^(^ ^8^ ^)^ chemotaxis,^(^ ^9^ ^)^ hepatitis C virus replication,^(^ ^10^ ^)^ and prolactin signaling.^(^ ^11^ ^)^

To better understand the physiological role of CypB, we previously generated *PpiB* ^*−/−*^ mice. Young mice lacking CypB developed OI, demonstrated by kyphosis, severe osteoporosis, and weight loss. Abnormal morphology of collagen fibrils and improper localization of procollagen were revealed in CypB‐deficient tissues and fibroblasts. At the molecular level, we found that CypB loss directly causes the destabilization of P3H1, which is essential for posttranslational prolyl‐3‐ hydroxylation of type I collagen.^(^ ^12^ ^)^

Although collagen abnormalities may directly contribute to the weakness of bones in patients with OI, they are also closely associated with accelerated and pathological aging. There is growing evidence for collagen cross‐linking, glycosylation, deficiencies, and mutations in alignment and distribution that promote the aging of skin, vasculature, kidney, and some other tissues.^(^ ^13^, ^14^, ^15^, ^16^ ^)^ Therefore, it is possible that some of the phenotypes in OI may result indirectly from accelerated aging, rather than solely through inadequate synthesis of properly folded collagen. Here, we have begun to test this concept through the use of CypB‐null mice, which is an established OI model lacking mutated collagen genes that might impact the results through abnormal molecular interactions. We find that genetic ablation of CypB induces aging‐associated cellular features and promotes premature aging phenotypes in mice. Loss of CypB leads to abnormal collagen and selective senescence of multipotent progenitor cells. Both factors trigger the upregulation of the senescence marker, p21, which is considered to be a probable driver in the cascade whereby CypB deficiency may accelerate organismal aging.

## Materials and Methods

### Animal studies

All animal trials were approved by the Mayo Clinic Institutional Animal Care and Use Committee.

All mice were housed under pathogen‐free conditions at 23°C to 25°C with a 12‐hour light/dark cycle and were fed with standard laboratory chow (PicoLab^R^ Rodent Diet 20; LabDiet, St. Louis, MO, USA; Cat# 0007688) with free access to water. The number of the mice per cage is five or lower than five. In‐cage shelter was provided for single‐housed mice. *PpiB* ^*−/−*^mice, generated by our laboratory, have exon 3 deleted by homologous recombination.^(^ ^12^ ^)^ The mice were bred on to C57BL/6 background (>10 backcrosses). To avoid the negative impact of abnormal teeth on feeding in *PpiB* ^*−/−*^ mice, we trimmed teeth once we spotted the abnormality during daily checkup. C57BL/6 (000664) mice were purchased from the Jackson Laboratory (Bar Harbor, ME, USA). p21 knockout mice^(^ ^17^ ^)^ were acquired from the Jackson Laboratory (016565) and were bred to *PpiB* ^*−/−*^ mice. The resulting double‐heterozygous mice were intercrossed to generate *PpiB* ^*−/−*^ mice carrying wild‐type, heterozygous, or null *p21* gene. One mouse from *p21* ^*+/−*^ *;PpiB* ^*−/−*^ group was removed from the study (Fig. 4*A*) due to severe skin lesion. Homozygous B6C3Fe *a*/*a*‐*Col1a2* ^*oim*^/J (*Col1a2* ^*oim*^, 00181) was purchased from the Jackson Laboratory. A minimum of *n* = 3 animals was used in most studies. Due to the limited availability of the mice, quantitative reverse‐transcription polymerase chain reaction (qRT‐PCR) analysis of senescence marker p16, p19, interleukin 6 (IL6), and p21 expression in inguinal adipose tissues (IAT) included two *Col1a2* ^*WT*^ and two *Col1a2* ^*oim*^ mice (Fig. 6*C*). All animal studies were performed in a double‐blinded manner.

### Body composition analysis

Abdominal subcutaneous and visceral adipose tissues were quantified at the third lumbar vertebrae in anesthetized mice by micro‐computed tomography (μCT) (vivaCT 40; Scanco Medical, Wayne, PA, USA) as described.^(^ ^18^ ^)^ Body composition of mice was assessed by noninvasive nuclear magnetic resonance spectroscopy (EchoMRI™‐100 System; Echo Medical Systems, Houston, TX, USA) allowing the quantification of fat and lean body mass.^(^ ^19^ ^)^ This noninvasive measure was performed on conscious mice.

### Histology

Skin and gastrocnemius muscle were dissected out from humanely euthanized 6‐month‐old *PpiB* ^*+/+*^ or *PpiB* ^*−/−*^ mice. Tissues were fixed in 10% formalin at room temperature for overnight, then processed in Leica ASP300S (Leica Biosystems, Deer Park, IL, USA) and embedded in paraffin using Leica EG1150C (Leica Biosystems). We cut 5‐μm sections of both tissues and stained them with hematoxylin and eosin following standard procedures.

### Generation and culture of bone marrow–derived multipotent stromal cells

Tibias and femurs were dissected from 6‐week‐old to 8‐week‐old *PpiB* ^*+/+*^ mice, *PpiB* ^*−/−*^ mice, or *p21* ^*−/−*^;*PpiB* ^*−/−*^ mice. The bone marrows were flushed out with serum‐free Dulbecco's Modified Eagle's Medium (DMEM; 1196‐092). After centrifugation, cells were plated with DMEM supplemented with 15% fetal calf serum (FBS) and penicillin/streptomycin and incubated at 37°C, 5% CO_2_ for 7–10 days without changing media. The adherent cells were then trypsinized, replated, and cultured for another round of 7–10 days without changing media. The subsequent growing adherent cells were considered as bone marrow–derived multipotent stromal cells and maintained in DMEM with 10% FBS.

### Generation and culture of primary osteoblasts

Calvarias were dissected from 6‐week‐old to 7‐week‐old *PpiB* ^*+/+*^ mice and *PpiB* ^*−/−*^ mice and were chopped into 1–2‐mm pieces. The bone pieces were washed with PBS and incubated with 2 mg/mL collagenase II solution (260 U/mg; Worthington Biochemical Corporation, Lakewood, NJ, USA; LS001474) at 37°C in a shaking water bath for 30 minutes. The collagenase II solution was discarded and replaced with fresh collagenase II solution for another 30‐minute incubation, followed by one round of 30‐minute incubation with 0.05 trypsin‐EDTA (Gibco, Grand Island, NY, USA; 25300‐054) and two consecutive rounds of 30‐minute incubation with collagenase II solution. The digested bone pieces were then rinsed and seeded in complete DMEM supplemented with 10% FBS and penicillin/streptomycin. Primary osteoblasts started to migrate out from the bone chips after 3–5 days of culture at 37°C, 5% CO_2_, 3% O_2_. The subconfluent cells, usually taking 11–15 days, were then trypsinized and replated in the new flasks. The bone pieces were left in the old flasks. The cells were used for experiments after approximately 7–10 days of further culture.

### Senescence‐associated beta‐galactosidase staining

Senescence‐associated beta‐galactosidase (SA‐β‐Gal) staining of mouse inguinal or visceral adipose tissue as well as primary multipotent stromal cells were performed using a kit according to the manufacturers' instructions (Cell Signaling Technology, Beverly, MA, USA; Catalog# 9860S). SA‐β‐Gal activity was identified based on positively stained blue cells. Mouse inguinal or visceral adipose tissues were excised, sectioned, and stored in PBS on ice until fixation. Adipose sections or cells were fixed in 1X Fixative Solution for 15 minutes at room temperature, washed twice in PBS, and developed in staining solution that contains the substrate X‐gal in a non‐CO_2_ chamber for 16 to 20 hours at 37°C. To quantify primary multipotent stromal cells stained for SA‐β‐Gal activity, we counterstained cells with Hoechst to visualize nuclei. The percentage of senescent cells was the total number of senescent cells divided by the total number of cells counted using immunofluorescence (*n* = 3 cell lines per genotype). All the quantitative analysis of the SA‐β‐Gal in IAT (Figs. 2*A*, 5*C*, and 6*B*) were performed by using Image J 1.53 k software (NIH, Bethesda, MD, USA; https://imagej.nih.gov/ij/).

### Quantitative real time–PCR

Total RNA was extracted from IAT using a Qiagen RNeasy plus mini kit (QIAGEN, Valencia, CA, USA; Catalog# 74136) according to the manufacturer's protocol. Transcription into complementary DNA (cDNA) was performed using Oligo‐dT and SuperScript III reverse transcriptase (Invitrogen, Carlsbad, CA, USA; Cat# 18080‐400) according to the manufacturer's instructions. All PCR reactions used SYBR green PCR Master Mix (Applied Biosystems, Foster City, CA, USA) to a final volume of 12 μL, with each cDNA sample performed in triplicate in the ABI PRISM7900 Sequence Detection System (Applied Biosystems) according to the protocol of the manufacturer. The expression of genes was normalized to 18S rRNA. Sequences of primers for 18S rRNA, p21, p19, IL6, and p16 were as follows: 18S rRNA forward: 5′‐CGCTTCCTTACCTGGTTGAT‐3′, 18S rRNA reverse: 5′‐GAGCGACCAAAGGAACCATA‐3′; p21 forward: 5′‐GTCCAATCCTGGTGATGTCC‐3′, p21 reverse: 5′‐GTTTTCGGCCCTGAGATGT‐3′; p19 forward: 5′‐GCCGCACCGGAATCCT‐3′, p19 reverse: 5′‐TTGAGCAGAAGAGCTGCTACGT‐3′; IL6 forward: 5′‐GACAACTTTGGCATTGTGG‐3′, IL6 reverse: 5′‐ATGCAGGGATGATGTTCTG‐3′; p16 forward: 5′‐ CCCAACGCCCCGAACT‐3′, p16 reverse: 5′‐ GCAGAAGAGCTGCTACGTGAA‐3′.

### Western blot analyses

Western blot analyses were carried out as described.^(^ ^20^ ^)^ Equal loading was determined using tubulin or actin. Antibodies for p21 (M‐19) were purchased from Santa Cruz Biotechnology (Santa Cruz, CA, USA; sc‐471). Antibodies for p53 (#2524) were purchased from Cell Signaling Technology (Danvers, MA, USA). Antibodies for p19 (NB200‐106) was purchased from Novus Biologicals (Littleton, CO, USA). Antibodies for CypB (ab16045) was purchased from Abcam (Cambridge, MA, USA). Membranes were visualized by developing films on Kodak X‐OMAT 2000A or by infrared imaging (Odyssey; LI‐COR Biosciences, Lincoln, NE, USA). We quantified p21 signal by the use of Image J 1.53 k software and normalized them to actin or tubulin.

### Faxitron X‐ray analysis

The mice were anesthetized with 2–3% isoflurane before imaging. The mice were positioned at the point where their curvatures were clearly seen. Images were taken on Faxitron LX‐60 (Faxitron X‐Ray LLC, Lincolnshire, IL, USA).

### Statistical analysis

All statistics were performed using GraphPad Prism 9 software (GraphPad Software, Inc., La Jolla, CA, USA). Data are expressed as medians with range.

## Results

### Loss of CypB leads to a progeroid phenotype in mice

**Fig. 1:**
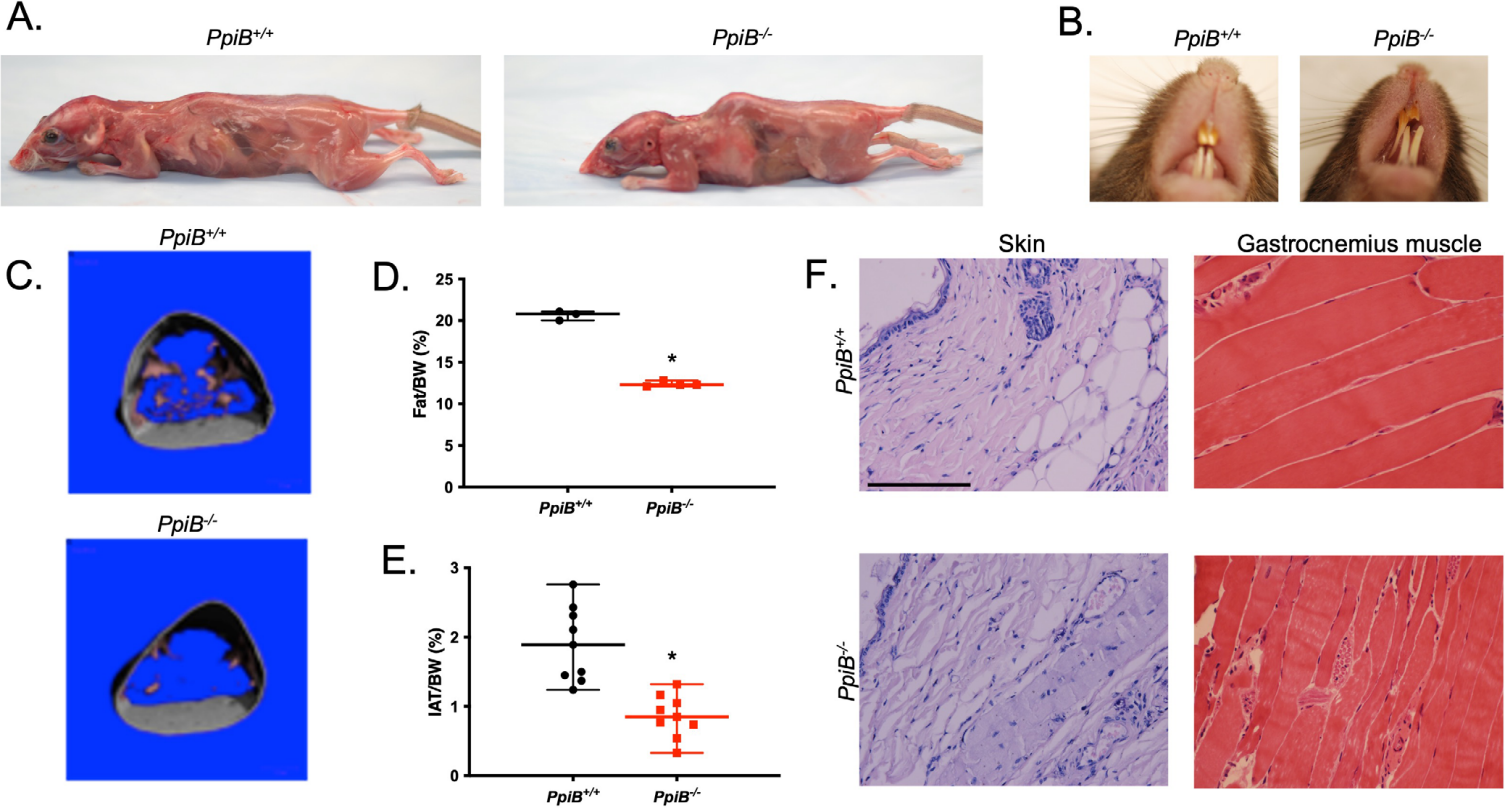
Premature‐aging phenotypes developed in CypB knockout mice. (*A*) Skinned 5‐month‐old *PpiB* ^*+/+*^ (left) and *PpiB* ^*−/−*^ (right) females. The *PpiB* ^*−/−*^ mouse had pronounced kyphosis. (*B*) Abnormal teeth observed in 1‐year‐old *PpiB* ^*−/−*^ mice. (*C*) Image of microcomputed tomography (Viva Computed Tomography 40 Scanner) for 4‐month‐old female *PpiB* ^*+/+*^ (left) and *PpiB* ^*−/−*^ (right) mice indicating the segmented visceral fat distribution. (*D*) Analysis of total body fat of *PpiB* ^*+/+*^ (*n* = 3) and *PpiB* ^*−/−*^ (*n* = 4) female mice at 8 months old using Echo‐MRI‐100 (*p < 0.01, unpaired two‐tailed *t* test). (*E*) Analysis of total IAT of *PpiB* ^*+/+*^ (*n* = 9; female = 5, male =4) and *PpiB* ^*−/−*^ (*n* = 9; female = 5, male =4) mice at 14–18 months old (*p < 0.01, unpaired two‐tailed *t* test). (*F*) H&E staining of skin and gastrocnemius muscle (longitudinal, 400×) in 6‐month‐old male *PpiB* ^*+/+*^ mouse (top) and *PpiB* ^*−/−*^ mouse (bottom). Scale bar = 100 μm. H&E = hematoxylin and eosin.

As reported earlier, CypB knockout mice are viable, though they have reduced body size and weight. They also develop thinner skin and severe osteopenia compared to littermate controls.^(^ ^12^ ^)^ The analysis by microcomputed tomography (μCT) revealed significantly reduced amounts of trabecular bone and reduced bone volume as well as increased separation between trabeculae in mice lacking CypB. The severity of osteopenia progressed along with aging. Consistent with previous findings, we observed profound kyphosis in young CypB knockout mice. Skinning of 5‐month‐old *PpiB* ^*−/−*^ mice confirmed their severe spinal kyphosis (Fig. 1*A*). At 8 months old, 50% of *PpiB* ^*−/−*^ mice, regardless of sex, developed kyphosis, whereas no age‐matched wild‐type mice had kyphosis. It has been known that more than 50% of OI patients have dentinogenesis imperfecta,^(^ ^21^, ^22^ ^)^ and we found that malocclusion developed in knockout mice starting at 2 months after birth, more often affecting the upper pair of incisors (Fig. 1*B*). The occurrence of the overgrowth of teeth in mice is strongly associated with advanced age. By 1 year of age, all knockout and no wild‐type mice developed such a deformity of their teeth. Given the possibility that abnormal teeth may interfere with eating, we trimmed teeth once we spotted the abnormality. We also examined food‐intake using Comprehensive Laboratory Animal Monitoring System. The result indicated no significant difference in food intake between CypB wild‐type and knockout mice after normalizing to their body weight (data not shown).

Another predominant feature of pathological aging is the loss of fat.^(^ ^23^, ^24^ ^)^ We examined the volume of visceral fat by μCT analysis in 4‐month‐old mice. As indicated in Fig. 1*C*, dramatically less visceral fat was seen in CypB knockout mice. To further explore the fat distribution, we used magnetic resonance imaging (MRI) to scan additional animals, normalizing the percentage of body fat to body weight. We also dissected IAT from these mice and calculated the ratio of IAT to body weight. Significantly less fat was observed in mice lacking CypB in both sets of analyses (Fig. 1*D*,*E*). Histological analysis of skin revealed a profound loss of collagen fibers beneath the dermis in 6‐month‐old CypB knockout mice. The subdermal adipocyte layer was also largely missing in those mice (Fig. 1*F*). Longitudinal sections of gastrocnemius muscle indicated more damage and degeneration in CypB knockout mice compared to wild‐type littermate controls. Taken together, we conclude that multiple phenotypes suggestive of aging‐associated features develop early in mice lacking cyclophilin B.

### CypB deficiency promotes SA‐β‐Gal activity in IAT and primary multipotent stromal cells

**Fig. 2:**
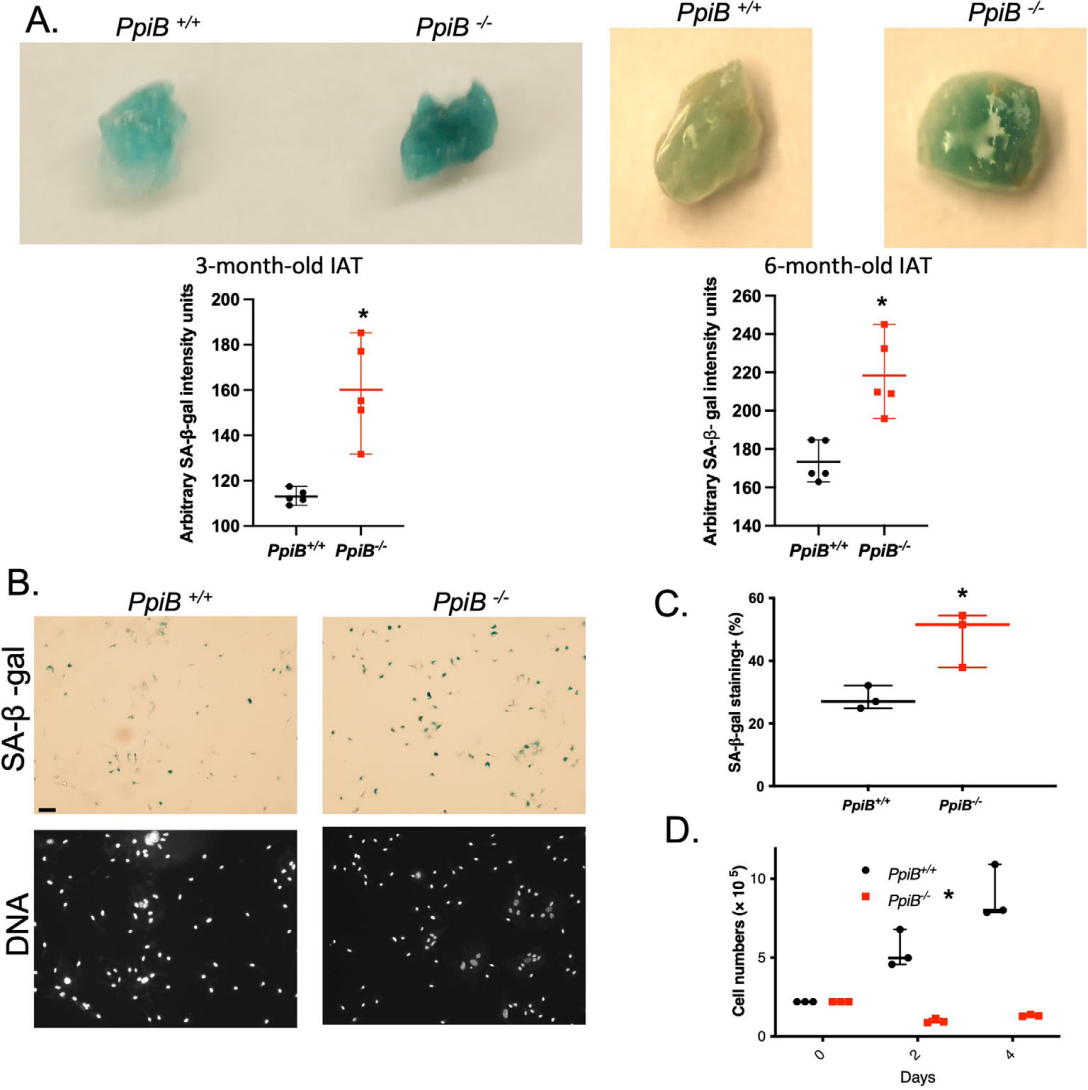
Profound SA‐β‐gal activities were observed in IAT and in primary multipotent stromal cells lacking cyclophilin B. (*A*) IAT of 3‐month‐old male with quantitative data for SA‐β‐gal activity. *PpiB* ^*+/+*^ (*n* = 5); *PpiB* ^*−/−*^ (*n* = 5), *p < 0.01, unpaired two‐tailed *t* test (left panel). IAT of 6‐month‐old female with quantitative data for SA‐β‐gal activity. *PpiB* ^*+/+*^ (*n* = 5); *PpiB* ^*−/−*^ (*n* = 5), *p < 0.01, unpaired two‐tailed *t* test (right panel). (*B*) SA‐β‐gal staining of primary multipotent stromal cells (200×). Scale bar = 100 μm. (*C*) Analysis of SA‐β‐gal activity of primary multipotent stromal cells generated from *PpiB* ^*+/+*^ (*n* = 3) and *PpiB* ^*−/−*^ (*n* = 3) mice, *p < 0.05, unpaired two‐tailed *t* test. (*D*) Analysis of cell growth of primary multipotent stromal cells at passage 5 (*PpiB* ^*+/+*^, *n* = 3; *PpiB* ^*−/−*^, *n* = 3; *p < 0.01, multiple *t* test).

Cellular senescence is considered to be an important contributor to aging in many tissues and organs.^(^ ^25^ ^)^ We therefore explored whether the progeroid phenotype observed in CypB‐deficient mice coincided with an increase in cellular correlates of senescence. IAT of 3‐month‐old and 6‐month‐old cyclophilin B knockout mice stained much more strongly for SA‐β‐Gal, than did those from age‐matched wild‐type mice (Fig. 2*A*). Such increase was consistently found in all IAT samples at both ages, raising the possibility that cyclophilin loss may indeed elicit cellular senescence. We then assessed whether loss of CypB induced markers of aging at the cellular level. We stained passage 5 primary multipotent stromal cells (MSCs) generated from bone marrow of wild‐type and *PpiB* ^*−/−*^ mice. Higher numbers of knockout cells were positive for SA‐β‐Gal activity (Fig. 2*B*,*C*). Cellular senescence is commonly associated with slower growth rate. In line with this, the growth of the knockout cells were significantly reduced compared to normal cells (Fig. 2*D*). Taken together, these data indicate that senescence might be promoted by depletion of CypB at both the cell and tissue levels.

### Senescence marker p21 is elevated in CypB‐deficient cells and tissues

**Fig. 3:**
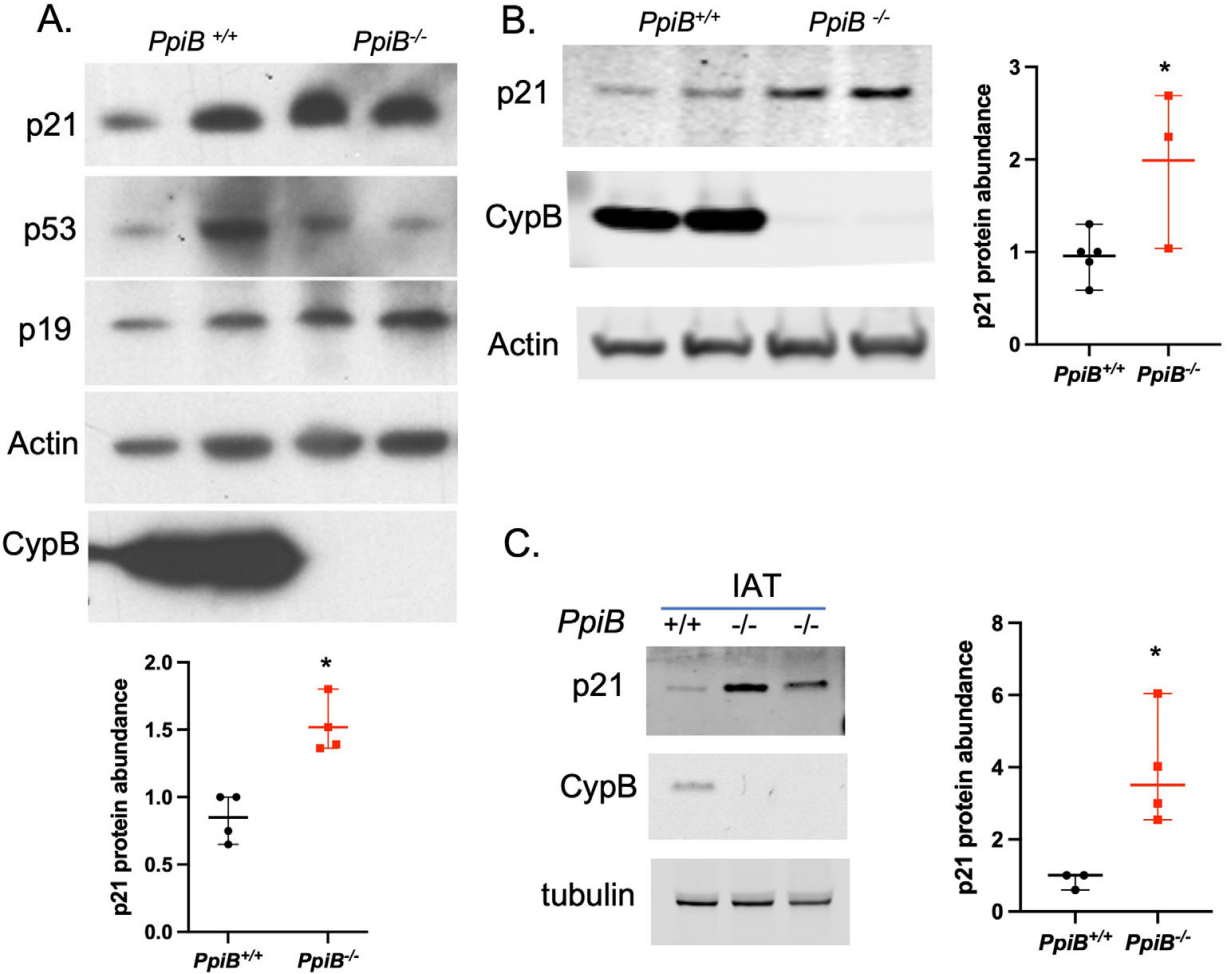
Senescence marker p21 protein is elevated in CypB‐deficient cells and tissues. (*A*) Western blot showing levels of p21, p53, p19, and CypB in wild‐type and CypB‐null primary multipotent stromal cells, with quantitative data of p21 from two independent experiments on two individual lines generated from each mouse strain. *p < 0.01, unpaired two‐tailed *t* test. (*B*) Western blot showing levels of p21 and CypB in wild‐type and CypB null primary osteoblasts with quantitative data of p21 from five *PpiB* ^*+/+*^ lines and three *PpiB* ^*−/−*^ lines. *p < 0.05, unpaired two‐tailed *t* test. (*C*) Western blot showing levels of p21 and CypB in IAT collected from CypB wild‐type (*n* = 3) and null mice (*n* = 4) and the quantitative data of p21 abundance in IAT. *p < 0.05, unpaired two‐tailed *t* test.

Though positive SA‐β‐Gal staining is a widely used biomarker for senescent and aging cells, there is variable expression of the assorted senescence markers in different cell types. To further ask if CypB loss induces additional features of senescence, we analyzed the expression of a series of markers, including p21, p16, p19, pai1, IL6, and insulin like growth factor binding protein 2 (Igfbp2), by qRT–PCR in IAT of 3‐month‐old and 6‐month‐old animals. No significant differences were observed among those markers (data not shown). Though not quite reaching statistical significance, p21 messenger RNA (mRNA) levels showed a trend to be upregulated in knockout tissues, compared to wild type. This finding led us to explore the levels of p21 protein in the absence of CypB. Given the possibility that the p53/p21 pathway may be involved in mediating cellular aging, we also examined the protein levels of p21, p53, and p19 in primary MSCs generated from bone marrow of knockout and wild‐type mice. Significantly, p21 protein was more highly abundant in CypB knockout cells than in wild‐type counterparts. Additionally, we found that p19 also accumulated more in cells lacking CypB. However, although p19 has been reported to modulate p53 levels, the latter were not elevated in CypB knockout cells, suggesting that the upregulation of p21 seen in CypB‐deficient cells and tissues was independent of p53 (Fig. 3*A*). We next examined p21 protein levels in primary osteoblasts and in IAT. Importantly, increased p21 protein levels were found in both cells and tissues from CypB knockout mice (Fig. 3*B*,*C*). This finding, together with elevated SA‐β‐Gal activity and slower growth rate in CypB‐deficient cells and tissues, suggests that cyclophilin loss may elicit features of cellular senescence.

### Loss of p21 delays the development of kyphosis in CypB‐deficient mice

**Fig. 4:**
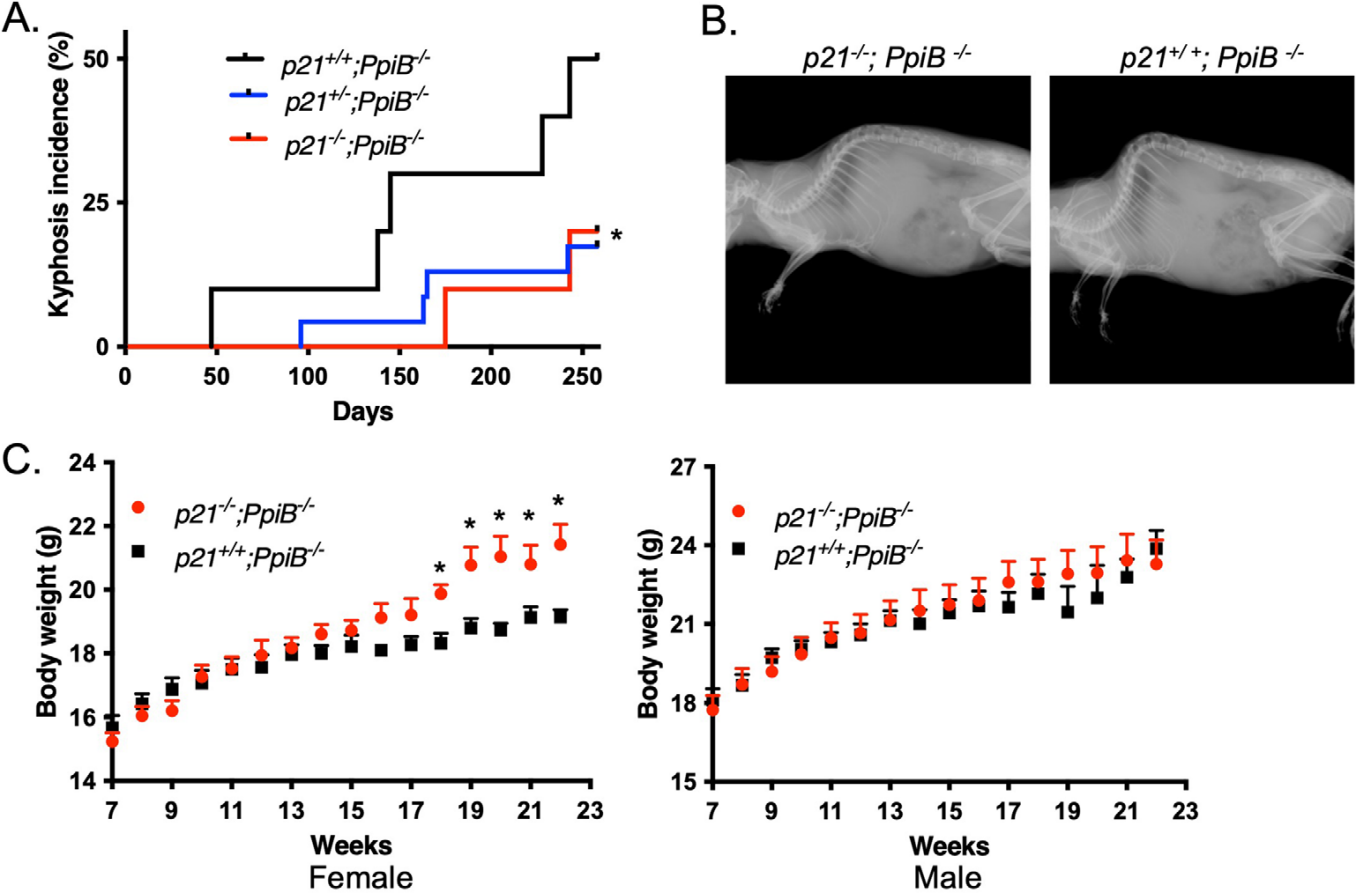
Partial recovery of *PpiB* ^*−/−*^ phenotype in a p21‐null background. (*A*) The kyphosis incidence in CypB‐deficient mice with *p21* ^*+/+*^ (*n* = 10), *p21* ^*+/−*^ (*n* = 23), or *p21* ^*−/−*^ (*n* = 10) background. *p < 0.05 between *p21* ^*+/+*^ *;PpiB* ^*−/−*^ group and the combined group of *p21* ^*+/−*^ *;PpiB* ^*−/−*^ and *p21* ^*−/−*^ *;PpiB* ^*−/−*^ (log‐rank test). (*B*) Faxitron X‐ray images of a *p21* ^*−/−*^ *;PpiB* ^*−/−*^ mouse and a *p21* ^*+/+*^ *;PpiB* ^*−/−*^ mouse. (*C*) Female p21 and CypB double KO mice had increased body weight. Male: *p21* ^*+/+*^ *;PpiB* ^*−/−*^ (*n* = 12); *p21* ^*−/−*^ *;PpiB* ^*−/−*^ (*n* = 13); female: *p21* ^*+/+*^ *;PpiB* ^*−/−*^ (*n* = 13); *p21* ^*−/−*^ *;PpiB* ^*−/−*^ (*n* = 16). *p < 0.05 at week 18, 19, 20, 21, and 22 in female mice (multiple *t* test).

Elevation of p21 levels could be an epiphenomenon of cellular senescence and progeroid defects in CypB knockout mice, or it could instead contribute to the phenotype. To discern between these possibilities, we asked whether any of the age‐associated phenotypes could be rescued by blocking abnormally increased p21 through breeding mice onto the p21‐homozygous null genetic background^(^ ^17^ ^)^ (all mice had the C57Bl/6 background). Because lordokyphosis and other age‐related phenotypes did not develop in *p21* ^*−/−*^ mice during the 1‐year observation period,^(^ ^26^ ^)^ we did not include *p21* ^*−/−*^ mice in this study. *PpiB* ^*−/−*^ mice carrying wild‐type p21 served as the comparison group. Cohorts of *p21* ^*+/+*^ *;PpiB* ^*−/−*^, *p21* ^*+/−*^ *;PpiB* ^*−/−*^, and *p21* ^*−/−*^ *;PpiB* ^*−/−*^ mice were monitored for the development of age‐related phenotypes for a period of up to 2 years. By examining the mice twice per week, we recorded the ages at which they were scored positive for kyphosis for two consecutive examinations. Consistent with our previous report,^(^ ^12^ ^)^ we found that CypB knockout mice began to develop kyphosis at 8 weeks. Remarkably, ablation of p21 delayed the onset of kyphosis to 14 weeks of age (Fig. 4*A*), though did not impact overall survival of *PpiB* ^*−/−*^ mice (data not shown). Interestingly, haploinsufficiency of p21 appeared to cause a similar improvement as did the complete loss of p21 in CypB‐deficient mice. The development of kyphosis was further demonstrated by faxitron X‐ray analysis, which revealed the typical underlying skeletal changes (Fig. 4*B*). These results suggested that p21 plays a critical role in the development of kyphosis due to loss of CypB. Echoing this finding, we saw a tendency to have higher bone volume and lower separation between trabeculae in mutant mice lacking both CypB and p21(data not shown). Besides the improvements in the bones, female mice lacking both p21 and CypB exhibited a faster gain of body weight at 18, 19, 20, 21, and 22 weeks of age compared to those of age‐matched *Ppib* ^*−/−*^ mice (Fig. 4*C*), suggesting that the general health of CypB null animals might also benefit from the removal of p21. Taken together, these results indicate that the absence of p21 partially improves some age‐related phenotypes observed in CypB‐KO mice.

### p21 depletion attenuates SA‐β‐gal activities in CypB knockout tissue and cells

**Fig. 5:**
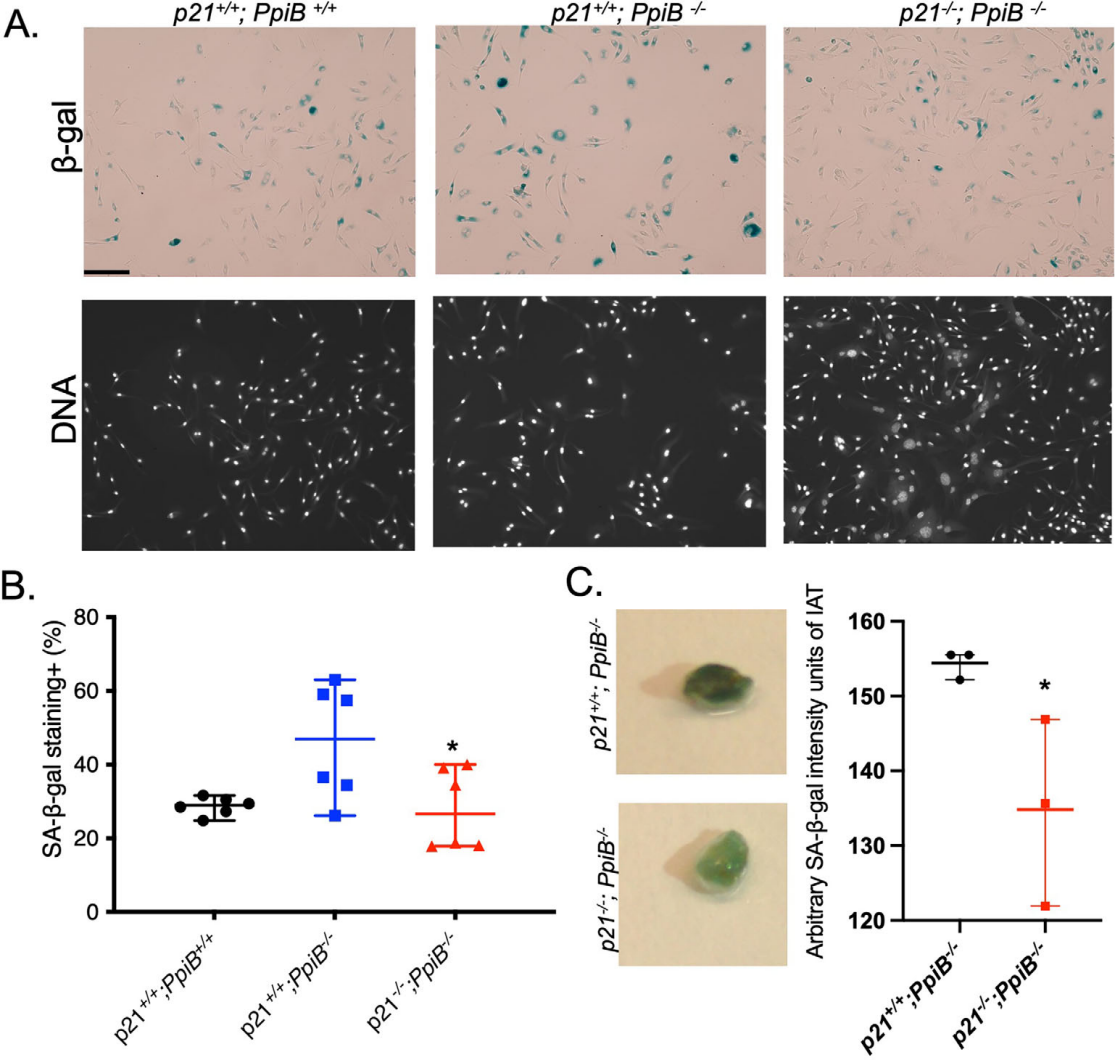
p21 loss attenuates SA‐β‐gal activities in CypB knockout tissue and cells. (*A*) Images of β‐gal staining on primary multipotent stromal cells (200×). The genotypes were indicated on the top. Scale bar = 100 μm. (*B*) Analysis of the rate with positive SA‐β‐gal staining in the primary multipotent stromal cells generated from *p21* ^*+/+*^ *;PpiB* ^*+/+*^, *p21* ^*+/+*^ *;PpiB* ^*−/−*^, and *p21* ^*−/−*^ *;PpiB* ^*−/−*^ mice (*p < 0.05, one‐way ANOVA test). (*C*) IAT of 3‐month‐old *p21* ^*+/+*^ *;PpiB* ^*−/−*^ and *p21* ^*−/−*^ *;PpiB* ^*−/−*^ mice stained for SA‐β‐gal activity with quantitative data. *p21* ^*+/+*^ *;PpiB* ^*−/−*^ (*n* = 3), *p21* ^*−/−*^ *;PpiB* ^*−/−*^ (*n* = 3), *p < 0.05, F test to compare variances.

We next investigated whether the absence of p21 impacts cellular senescence features. Passage 5 primary MSCs generated from wild‐type, *p21* ^*+/+*^ *;PpiB* ^*−/−*^, or *p21* ^*−/−*^ *;PpiB* ^*−/−*^ mice were stained with SA‐β‐gal. Significantly fewer β‐gal–positive cells were observed in p21 and CypB double KO cells, compared to cells lacking only CypB (Fig. 5*A*,*B*). We also stained the IAT collected from 3‐month‐old mice. The adipose tissue samples in the *p21* ^*−/−*^ *;PpiB* ^*−/−*^ exhibited significantly less profound SA‐β‐gal staining than the same tissues in *p21* ^*+/+*^ *;PpiB* ^*−/−*^ mice (Fig. 5*C*). Taken together, these results indicate that p21, downstream of CypB‐loss, may plays a role in the progression of accelerated aging‐associated features in CypB‐KO mice.

### Collagen mutant *Cola2^oim^* mice carry some premature‐aging related features

We next asked whether these phenotypes reflect a specific role for CypB in suppression of age‐associated cellular characteristics, or instead is a general feature of OI. We employed *Col1a2* ^*oim*^ mice, a well characterized model for human OI that results from expression of a spontaneously mutated Col1a2 pro‐alpha 2 collagen gene. Homozygous B6C3Fe *a*/*a*‐*Col1a2* ^*oim*^/J mice exhibit osteopenia, progressive skeletal deformities, cortical thinning, and small body size.^(^ ^21^ ^)^ To our knowledge, they have not been studied by others for evidence of accelerated aging or features of cellular senescence.

**Fig. 6:**
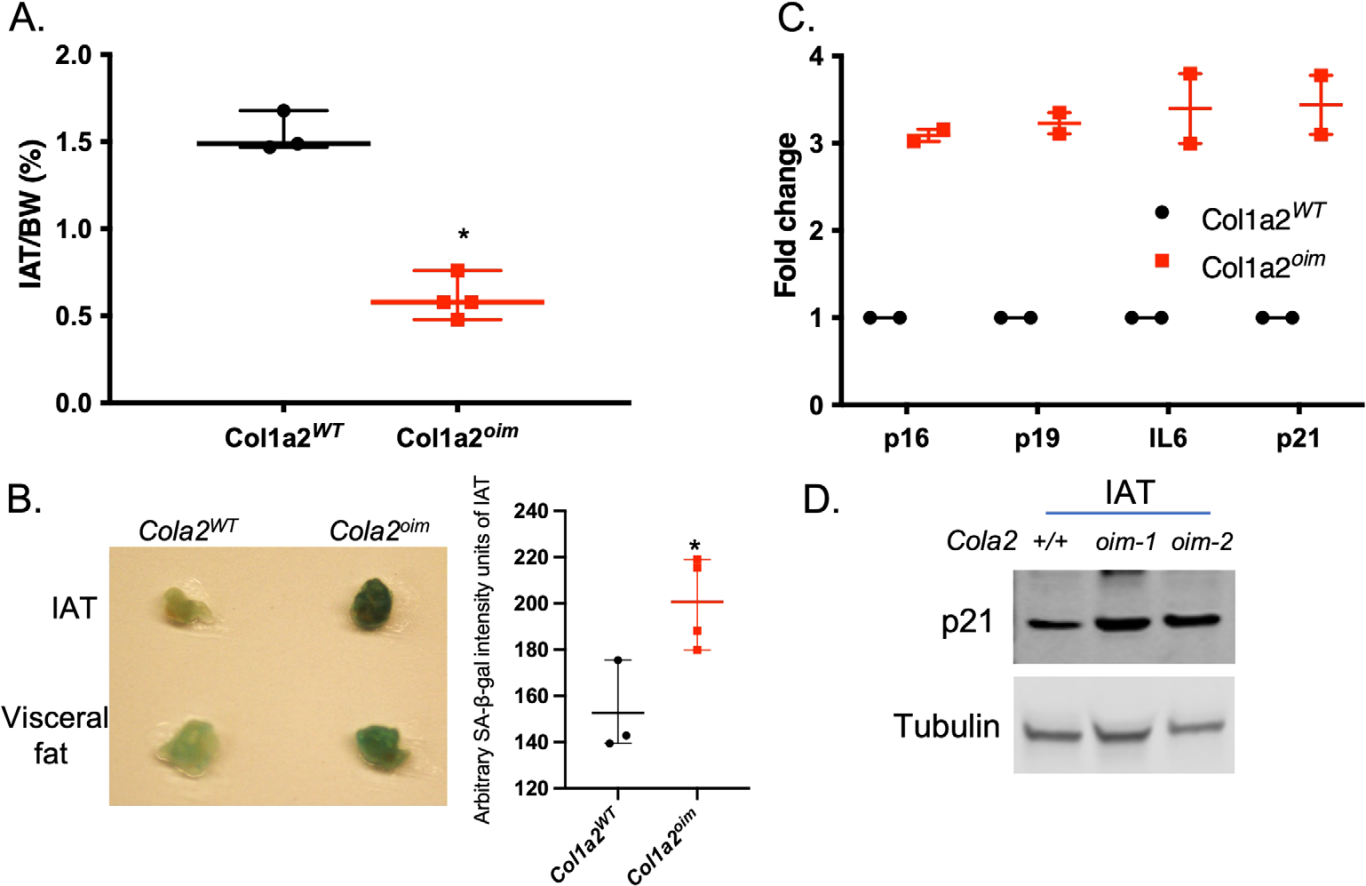
Collagen mutant *Col1a2* ^*oim*^ mice carry some premature‐aging associated phenotype. (*A*) The collagen mutant mice (3‐month‐old) have significantly reduced fat deposits. The percentage of IAT to the body weight (*n* = 3 in *Col1a2* ^*WT*^ group; *n* = 4 in *Col1a2* ^*oim*^ group; *p < 0.01, unpaired two‐tailed *t* test). (*B*) IAT and visceral fat of 3‐month‐old *Col1a2* ^*WT*^ and *Col1a2* ^*oim*^ mice stained for SA‐β‐gal activity with quantitative data of IAT. *Col1a2* ^*WT*^ (*n* = 3); *Col1a2* ^*oim*^ (*n* = 4); *p < 0.05, unpaired two‐tailed *t* test. (*C*) qRT‐PCR analysis of senescence marker p16, p19, IL6, and p21 expression in IAT (*n* = 2 per group). (*D*) Western blot analysis of p21 levels in IAT of 3‐month‐old *Col1a2* ^*WT*^ and *Col1a2* ^*oim*^ mutant mice (*n* = 2 per group).

We first dissected IAT from 3‐month‐old mice and calculated the ratio of the IAT to the body weight. Significantly less fat was observed in this analysis (Fig. 6*A*) for the *Col1a2* ^*oim*^ mice compared to *Col1a2* ^*WT*^ mice. IAT and visceral fat from both types of mice were stained for SA‐β‐gal activity. More profound SA‐β‐gal staining was found in *Col1a2* ^*oim*^ tissues (Fig. 6*B*). We further examined the senescence markers p16, p19, IL6, and p21 expression in IAT. Due to discontinued supply of homozygous *Col1a2* ^*oim*^ mice, we only had two mice of each genotype in this study. Interestingly, all markers were higher in *Col1a2* ^*oim*^ cells (Fig. 6*C*). p21 protein levels in mutant IAT was also higher than wild‐type counterpart (Fig. 6*D*). Taken together, the current results suggest that the *Col1a2* ^*oim*^ mutant mice recapitulate some findings from CypB knockout mice indicating a likely early onset of cellular senescence and aging in mice through a pathway connected with p21 upregulation, though more mice will need to be included in an expanded study when they become available, in order to solidify this conclusion.

## Discussion

In this study, we newly identified a number of progeroid phenotypes in *PpiB* knockout mice, including abnormal teeth, reduced adiposity, and sarcopenia. These data, together with increased SA‐β‐gal activity and elevated levels of the senescence marker p21, lead to the novel conclusion that loss of CypB may accelerate pathological aging. A growing list of genes that play important roles in regulation of DNA damage repair, telomere dynamics, nuclear integrity, homeostasis of MSCs, and mitotic checkpoint has been revealed to link to human progeria syndromes or mouse models that develop premature aging.^(^ ^27^ ^)^ This study adds the first gene that encodes a peptidyl‐prolyl isomerase, CypB, to this list.

In this work, we first used SA‐β‐gal to examine the incidence of premature senescence in murine embryonic fibroblasts (MEFs, we intercrossed CypB heterozygous mice to derive MEFs from individual 13.5‐day‐old fetuses). Surprisingly, we did not see significant differences between primary wild‐type and *PpiB* ^*−/−*^ MEFs in vitro (data not shown). In contrast, profound SA‐β‐gal staining was found in cultured bone marrow–derived MSCs generated from null animals. These results suggested that the sensitivity of SA‐β‐gal assay could be varied by cell type. As indicated by a number of literatures,^(^ ^28^, ^29^, ^30^ ^)^ it would be more reliable to access cellular senescence and aging by combining SA‐β‐gal assay with additional signature features of senescent cells, such as morphological change, lacking proliferation markers, or activating tumor‐suppressor network. Therefore, we further looked cell growth rate and protein levels of senescent marker p21 in primary MSCs lacking CypB. In line with higher β‐gal activity, we found slower growth and more p21 in CypB knockout MSCs. Together, these findings raised a possibility that insufficiency of CypB may preferentially promote senescence in this specific cell‐type, including the progenitor cells residing in animal tissues. This will help to explain the premature aging phenotype carried by null mice because senescence of multipotent precursor cells could deplete the progenitor cell pool and perturb subsequent growth or differentiation of derivative cells such as osteoblasts, chondrocytes, and adipocytes. Overall, we believe that selective senescence of multipotent precursor cells and abnormal collagen could contribute to aging in *PpiB* null animals. However, whether abnormal collagen can directly accelerate senescence of multipotent precursor cells remains to be further investigated.

A critical downstream factor that drives age‐associated phenotypes in the context of CypB loss is the cell cycle inhibitor p21. Elevated p21 was found in CypB KO–derived cells and tissues. Absence of p21 alleviated SA‐β‐gal staining and rescued portions of the age‐related phenotypes such as kyphosis, observed in CypB knockout mice. We also observed less fat deposit, higher SA‐β‐gal activity, and a trend toward p21 upregulation in *Col1a2* ^*oim*^ mice, suggesting this as a common feature of OI. It will be very helpful for further establishment of the connection between p21 and OI by expanding the studies on more *Col1a2* ^*oim*^ mice or other OI mouse models.

A gradual overall reduction of collagen mass occurs during normal aging. Abnormalities of collagen amount or posttranslational modifications have also been postulated to be possible contributors to aging, rather than simply a result of this inexorable process. Glycation of collagen and other extracellular matrix proteins have been observed in aged animals, and were proposed to contribute to age related pathologies.^(^ ^31^ ^)^ Furthermore, a study using mice that carry a mutated collagenase‐resistant type I collagen gene (*Col1a1r/r*) demonstrated that aberrant collagen directly drives the development of premature aging by reducing replicative lifespan and promoting cellular senescence.^(^ ^15^ ^)^ These mice also had elevated levels of p21; however, it was not determined in that report whether or not p21 played a role in age‐related phenotypes of that strain. In the current study, we explored *Col1a2* ^*oim*^ mouse model that expresses a mutated collagen which blocks the association of pro‐alpha 2 with the pro‐alpha1 chains. We found less fat deposits and more profound SA‐β‐gal staining in these mice. Interestingly, expressions of several senescent markers and p21 protein levels were trending upward. These results, together with the findings in our CypB knockout mice, favor the hypothesis that abnormal collagen is a driver of organismal aging in these models. However, we cannot completely rule out the possibility that CypB has an alternative or additional client substrate that contributes to its impact on senescence. Indeed, a proteomics analysis^(^ ^32^ ^)^ of lysates from CypB‐deficient dermal fibroblasts revealed dramatic reductions in lamin A, a protein known to underlie the progeria disease Hutchinson‐Gilford syndrome (HGS). Thus, future studies should investigate whether ER stress resulting from misfolded collagen might initiate cellular senescence indirectly through impaired posttranslational processing of other ER proteins, such as lamin A. Consistent with that, our previous study demonstrated that CypB loss induces ER an abnormal unfolded protein response (UPR, a celluar stress response related to ER stress).^(^ ^33^ ^)^ With the notion that UPR function is impaired with age,^(^ ^34^ ^)^ the clarification of the relationship among CypB, lamin A/C, and ER stress in the future will refine our understanding on severe OI and the progeria driven by CypB loss.

The role of p21 in aging is highly complex. It has been reported that p21 drives cellular senescence and age‐related pathology in multiple progeroid mouse models. Upregulation of p21 in intestinal progenitor cells and hematopoietic stem cells as well as higher numbers of SA‐β‐Gal–positive cells was observed in aging telomere dysfunctional mice (*mTR* ^*−/−*^ and *Terc* ^*−/−*^) that lacked telomerase activity.^(^ ^35^ ^)^ Deletion of p21 extended the lifespan of these mice because p21 deficiency significantly improved the stemness of multipotent progenitor cells with dysfunctional telomeres.^(^ ^36^ ^)^ MEFs from progeroid mice that carry low levels of the spindle assembly checkpoint protein BubR1 also had elevated p21 and increased cellular senescence.^(^ ^37^ ^)^ Ablation of p21 delayed senescence‐dependent cataract formation in BubR1 hypomorphic mice. On the other hand, p21 loss further elevated the senescence of the progenitors in skeletal muscle and fat, thus accelerating the degeneration of these tissues in BubR1 insufficient mice.^(^ ^26^ ^)^ Together, these findings reveal that p21 has dual roles in the aging process. In most cases, p21 promotes cellular senescence and advances aging; whereas in the context of certain circumstances such as BubR1 insufficiency, which may vary by tissue or cell type, p21 can inhibit aging by preventing cellular senescence. This may help to explain why we saw only partial rescue in *p21* ^*−/−*^ *;PpiB* ^*−/−*^ double knockout mice. As a cell cycle inhibitor, activation of p21 can protect cells from stresses or damage by slowing or blocking proliferation. Reduction of p21 would remove this layer of protection, which may ultimately be detrimental. In order to determine the net role of p21 during aging, it will be helpful to examine the status of cells that re‐enter the cell cycle with either normal or damaged DNA, or that become senescent or quiescent after cell arrest. In the context of chronic and persistent damage, p21 activation is likely to be disadvantageous.

As a genetic bone disorder, OI occurs at birth and is maintained lifelong. Though surgical care of fractured bones, pain medication, and physical therapy help to manage the disease, there is not yet a cure for OI. Our finding that removal of p21 delays the development of kyphosis in CypB‐deficient mice raises the notion that p21 inhibitors might aid in the treatment of OI, although clearly its role in tumor suppression must not be ignored. In addition, our study suggests that cell senescence may contribute to the severity of pathogenic features in OI. In the future as clinical use of senolytics advances, we believe that OI will be added to the list of diseases that may benefit from selective depletion of senescent cells.

## Author Contributions

**Ying Zhang:** Data curation; formal analysis; investigation; methodology; project administration; writing – original draft; writing – review and editing. **Robert J Pignolo:** Methodology; writing – review and editing. **Richard J Bram:** Conceptualization; data curation; funding acquisition; supervision; validation; writing – review and editing.

## Conflicts of Interest

No conflicts of interest, financial or otherwise, are declared by all authors.

### Peer Review

The peer review history for this article is available at https://publons.com/publon/10.1002/jbm4.10674.

## Data Availability

The data that support the findings of this study are available from the corresponding author on reasonable request.
